# Simultaneous assessment of mitochondrial and vascular function using the Flow Mediated Skin Fluorescence technique

**DOI:** 10.3389/fphys.2025.1509159

**Published:** 2025-02-19

**Authors:** Andrzej Marcinek, Joanna Katarzynska, Jerzy Gebicki

**Affiliations:** ^1^ Institute of Applied Radiation Chemistry, Lodz University of Technology, Lodz, Poland; ^2^ Angionica Ltd., Lodz, Poland

**Keywords:** FMSF technique, NADH fluorescence, vascular circulation, mitochondrial dysfunction, microcirculatory oscillations

## Abstract

Flow Mediated Skin Fluorescence (FMSF) is a new non-invasive diagnostic method for assessing vascular circulation and/or metabolic regulation. The method measures stimulation of the circulation in response to post-occlusive reactive hyperemia (PORH). It analyzes the dynamical changes in NADH fluorescence emitted from skin tissue, providing information on mitochondrial metabolic status and intracellular oxygen delivery through the circulatory system. Assessment of the vascular state using the FMSF technique is based on three parameters: flowmotion (FM) under normoxia conditions, hypoxia sensitivity (HS), and hyperemic response (HR_max_). The functioning of mitochondria can be assessed by analyzing the ischemic response (IR_max_), hypoxia sensitivity (HS), and the basal level of NADH fluorescence. There is a close relationship between the functioning of mitochondria and the vascular system. Despite these interactions, mitochondrial and vascular regulatory function can be monitored separately as well as simultaneously by the FMSF technique. Uniquely, this approach delivers information on both mitochondrial and vascular function based on a single measurement.

## 1 Introduction

Flow Mediated Skin Fluorescence (FMSF) is a novel non-invasive diagnostic technique based on the measurement of nicotinamide adenine dinucleotide (NADH) fluorescence from skin tissue cells. The diagnostic potential of the method is based primarily on stimulation of the circulation in response to post-occlusive reactive hyperemia (PORH). This is the most popular test used to assess vascular reactivity in both macro- and microcirculation. Occlusion of the brachial artery induces reactive hyperemia. Several important mediators are involved, with nitric oxide (NO) acting as a potent vasodilator of muscle-type arteries (Roustit and Cracowski, 2013). The progress and treatment of various diseases associated with vascular dysfunction can be monitored.

NADH and its oxidized form (NAD^+^) play a crucial role in biological systems as redox coenzymes (Gebicki et al., 2004). Under normal aerobic conditions, NADH is oxidized to NAD^+^ in mitochondria. During hypoxia, insufficient oxidation leads to excessive NADH accumulation. During hyperemia accelerated oxidation leads to excessive NADH depletion. These processes lead to different balances of NADH/NAD^+^. Therefore, the NADH/NAD^+^ couple can be treated as a sensitive marker of changes in both mitochondrial function and vascular perturbations responsible for the transport of oxygen and other nutrients to cells. Thus, measuring NADH fluorescence should enable assessment of both vascular circulation and metabolic regulation.

As the name of the method itself suggests, the FMSF technique measures NADH fluorescence from the skin, mainly on the forearm. The fluorescence of NADH is the strongest component in the overall fluorescence emitted from human skin and the skin is the most easily accessible measurement site. The AngioExpert, a device constructed by Angionica Ltd., measures NADH fluorescence (460 nm) excited by a 340 nm ultraviolet light at a sampling frequency of 25 Hz (Katarzynska et al., 2019b). The methodological aspects of the method (the source of the signal - NADH, reproducibility of measurements) were investigated (Bugaj et al., 2019; Hellmann et al., 2017; Tarnawska et al., 2018). The penetration of exciting light in skin tissue is low (about 0.3–0.5 mm) and a substantial fraction is absorbed by the epidermis and papillary dermis (Cicchi et al., 2013). The NADH signal is not disturbed by the presence of blood vessels (Hou et al., 2022).

Mayevsky’s pioneering work first drew attention to the potential of using NADH fluorescence to determine mitochondrial function *in vivo* (Mayevsky and Barbiro-Michaely, 2009; Mayevsky and Rogatsky, 2007). NADH fluorescence and mitochondrial dynamics have also been used to study the metabolism of keratinocytes in human skin (Balu et al., 2013). Although the keratinocytes have lower mitochondrial density than other cells, the mechanisms of NADH accumulation during ischemia and of NADH oxidation during hyperemia may reflect the general mechanism of mitochondria functioning in the cells of other organs (Pawlak-Chomicka et al., 2023; Ronquist et al., 2003; Waddell et al., 2023). Numerous other studies have shown that the skin microcirculation reflects the systemic microcirculation, its dysfunction, and pathologies (Cracowski and Roustit, 2020). This makes skin cells a sensitive marker of early disorders of vascular circulation, as it is possible to test microvascular blood circulation via changes in skin biochemistry–especially the mitochondrial NADH redox state (as an internal marker) of epidermal cells, which depends on blood circulation and is sensitive to its changes. This approach should allow the monitoring vascular dysfunction, assuming proper mitochondrial functioning. It should also enable monitoring of mitochondrial dysfunction, assuming proper functioning of the circulatory system. Obviously, there may be some overlap in the perturbations related to these factors.

To date, most studies on the FMSF technique have concentrated on the assessment of vascular circulation (Gebicki et al., 2021; Katarzynska et al., 2022; Marcinek et al., 2024a; Marcinek et al., 2024b; Mikosiński et al., 2023). The FMSF method was compared with other microcirculation assessment techniques using PORH, like, for example laser Doppler flowmetry (LDF), laser speckle contrast imaging (LSCI), flow mediated dilation (FMD), reactive hyperemia peripheral arterial tonometry (RH-PAT) (Liu et al., 2024; Szczepanek et al., 2024).

In this paper we focus on the application of the FMSF technique in assessing mitochondrial function. We provide examples illustrating vascular and mitochondrial dysfunctions in the distinct phases of FMSF measurement, including baseline collection, ischemia, and hyperemia with reperfusion.

## 2 Brief description of the analyzed groups

The analysis involved two patient groups: one with vascular diseases (CVD) and the other with type 2 diabetes (DM2), as described in previous publications (Katarzynska et al., 2020; Mikosiński et al., 2023). Brief clinical characteristics of the analyzed groups are presented in the Supplementary Material.

## 3 New diagnostic perspective of the FMSF technique

### 3.1 NADH fluorescence basal level

During the initial stage of FMSF measurements, the level of NADH fluorescence is determined.

#### 3.1.1 Mitochondrial function

The level of basal fluorescence (FL_base_) is expected to be related to the overall NADH/NAD^+^ redox balance, providing at minimum insight into whether this balance in specific diseases is shifted towards reduction (increase in NADH fluorescence) or oxidation (decrease in NADH fluorescence). The validity of this approach can be confirmed by comparing different patient groups. For example, in the group of patients with DM2, the basal fluorescence was shifted towards reduction (increase of NADH fluorescence) compared to the group of patients with CVD (Table 1) (see also: Wu et al., 2016; Yan, 2021). In both patient groups, women showed higher basal fluorescence, although the difference was not always statistically significant (Supplementary Table S1). Significantly higher levels of NADH fluorescence have been observed for patients with mitochondrial disease and m.3243A>G mutation confirmed in genetic testing (van Kraaij et al., 2023). Other examples also seem to confirm this differentiation–e.g. in the case of patients with psoriasis the NADH fluorescence level in psoriatic lesions was significantly reduced (decreased fluorescence) (Gebicki et al., 2023a). In contrast, it has been shown in the case of competitive athletes strenuous physical exertion (exertion to exhaustion) can significantly shift the NADH level towards reduction (increased fluorescence) (Bugaj et al., 2019; Bugaj et al., 2020).

**TABLE 1:** Analysis of the FMSF parameters for CVD and DM2 groups. Results are shown as mean ± standard deviation (SD) with corresponding *p*-values indicating only statistically significant differences (*p* < 0.05). Reproduced from Mikosiński et al. (2023), Katarzynska et al. (2020).

	CVD (N = 482)			DM2 (N = 70)		
	IR_max_ > 0	IR_max_ < 0	*p-value*	IR_max_ > 0	IR_max_ < 0	*p-value*
N	320	162		46	24	
Female/Male	163/157	101/61		18/28	14/10	
Age [years]	69.2 ± 10.7	69.7 ± 10.7		62.1 ± 8.4	65.0 ± 7.2	
BMI [kg/m^2^]	28.8 ± 5.8	30.6 ± 6.1	0.002^a^	29.9 ± 4.6	34.3 ± 6.3	0.005^b^
FL_base_ × 10^3^ [a.u.]	474 ± 213	500 ± 191		657 ± 277	822 ± 328	0.014^a^
log(FM)	1.33 ± 0.41	1.32 ± 0.39		1.46 ± 0.35	1.46 ± 0.30	
IR_max_ [%]	11.2 ± 5.7	—^c^		11.5 ± 4.6	—^c^	
HR_max_ [%]	15.5 ± 5.1	17.9 ± 4.8	<0.0001^a^	15.6 ± 4.4	17.3 ± 4.3	
log(HS)	1.19 ± 0.54	0.91 ± 0.52	<0.0001^a^	1.42 ± 0.47	1.10 ± 0.47	0.010^b^

^a^ Mann-Whitney test.

^b^ t-test.

^c^ numerical values not calculated for all cases studied.

Abbreviations: BMI, body mass index; FL_base_, fluorescence at baseline; log (FM), flowmotion at baseline (logarithm); IR_max_, Ischemic Response; HR_max_, Hyperemic Response; log (HS), Hypoxia Sensitivity (logarithm).

Unfortunately, the level of baseline fluorescence varies widely among homogeneous groups of patients, depending on skin pigmentation, suntan, and skin lesions. This introduces a significant error when the NADH/NAD^+^ balance and mitochondrial function are interpreted based solely on NADH fluorescence. Additional calibration methods are therefore required, both for the measurement method and for the patient’s skin type and condition.

### 3.2 Baseline collection

The FMSF signal is collected for 3 min and normalized with respect to the mean fluorescence value. Normalization of the signal makes the result independent of measurement conditions related to the individual characteristics of the patient’s skin and/or different technical factors, as only relative changes are analyzed.

#### 3.2.1 Microcirculatory function

The normalized fluorescence signal at the baseline, which characterizes the resting flow, is collected using the FMSF method over a period of 3 min. There are no strict time constraints, other than the patient’s comfort during the measurement, which requires immobility. The signal typically shows slight intensity changes, resulting from microcirculation oscillations–also called flowmotion (Aalkjær et al., 2011; Bernjak et al., 2008; Gebicki et al., 2020; Marcinek et al., 2023; Nilsson and Aalkjaer, 2003). The parameter FM (flowmotion), which can be a simple measure of these oscillations, is based on assessment of oscillations in terms of the mean square deviations of the experimental signal (at a sampling frequency of 25 Hz) from the baseline. Whereas the FM parameter varies within quite a broad range, log (FM) remains normally distributed.

The strength of oscillations contained in the FMSF signal can be determined using the Fast Fourier Transform (FFT) algorithm. The oscillations are grouped into three frequency intervals, corresponding to endothelial, neurogenic, and myogenic activities, respectively: ≤0.021 Hz, (0.021–0.052 Hz), and (0.052–0.15 Hz) (Bernjak et al., 2008; Gebicki et al., 2020; Marcinek et al., 2023). Comparison of FM parameters allows for the assessment of microcirculation functioning in different patients (Gebicki et al., 2020). A significant reduction in the FM parameter indicates microvascular dysfunction. Analysis of the components of the oscillation and their interrelationships allows for the diagnosis of conditions including extreme fatigue in athletes and post-COVID fatigue (with statistically significant short-term or long-term reductions in endothelial oscillations, respectively) (Chudzik et al., 2022). Relative reductions or increases of myogenic oscillations can be used to assess stress of various origins (Gebicki et al., 2023b; Marcinek et al., 2023).

### 3.3 Ischemic response

In the second stage of measurements, known as the ischemic response (IR), a change in NADH fluorescence is observed due to occlusion of the brachial artery by a cuff inflated to 60 mmHg above the systolic blood pressure of the patient.

#### 3.3.1 Mitochondrial dysfunction

Since blood supply is completely blocked at this stage, the fluorescence signal does not have the characteristics of blood flow (the signal remains completely smooth, there are no visible microcirculation oscillations, including in the cardiac component). The ischemic response can be entirely attributed to a change in cellular oxygen metabolism from an aerobic process to anaerobic processes (including glycolysis). Any disturbances in the ischemic part can be attributed to mitochondrial dysfunction. The use of FMSF-PORH method to assess mitochondrial functioning seems to be a perspective approach, due to the fact that most methods using PORH remain “blind” during the occlusion period.

**FIGURE 1:**
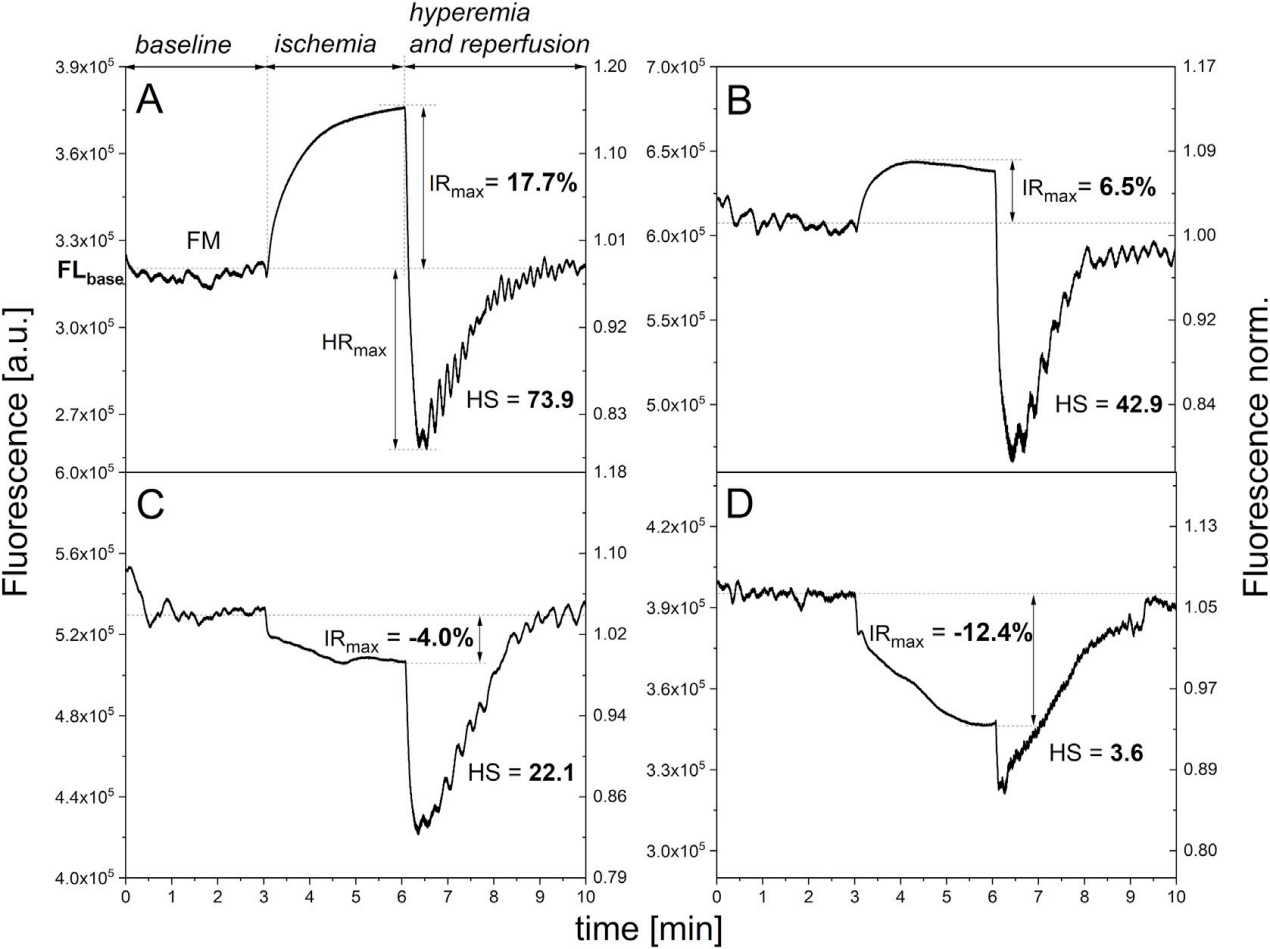
Exemplary FMSF traces showing different levels of mitochondrial function: **(A)** adequate mitochondrial function (female, 64 y.); **(B)** slightly impaired mitochondrial function (female, 77 y., diabetes, hypertension, cardiovascular disease); **(C)** impaired mitochondrial function (female, 69 y. diabetes, hypertension, cardiovascular disease); **(D)** Significantly impaired mitochondrial function (female, 65 y. hypercholesterolemia, hypertension, cardiovascular disease, heart failure).

The typical kinetics of ischemic changes, which are particularly characteristic for volunteers with no diagnosed health problems, is presented in Figure 1A. Such signal waveforms are also observed for some patients with diagnosed diseases, including diseases associated with mitochondrial dysfunction. After approximately 3 min or less, the signal stabilizes. The IR_max_ parameter, which is a measure of the ischemic response, corresponds to the maximum change in NADH fluorescence occurring at this stage (usually after 3 min of occlusion). This value typically reaches several percent relative to the baseline fluorescence, although in some cases it can reach over 20 percent. In patients suffering from various diseases, it typically reaches much lower values (Table 1).

However, deviations from a typical adequate ischemic response are often observed in this range. In some cases, the change in NADH fluorescence is weaker (Figure 1B), or it slightly deviates from the baseline before occlusion indicating impaired ischemic response (Figure 1C). However, sometimes the fluorescence signal, and therefore the concentration of the reduced NADH form, drops by several percent, almost as a mirror image of the typical signal course. In such cases, we can speak of a negative, abnormal response to occlusion (significantly impaired ischemic response) (see Figure 1D). Additionally, changes in the shape of the signal course are often observed, as well as some irregularities. The course remains characteristic for a given patient (Mikosiński et al., 2023; Pawlak-Chomicka et al., 2023).

Although the mechanisms of atypical ischemic responses–such as unexplained changes in the transition from oxidative phosphorylation to anaerobic glycolysis, changes in the share of mitochondrial and cytoplasmic NADH in fluorescence, and/or changes in the mitochondrial lactate metabolism (Glancy et al., 2021) –remain unclear, atypical FMSF signal patterns may be the result of impaired metabolic processes associated with the loss of or altered mitochondrial functionality (Pawlak-Chomicka et al., 2023). These changes cannot be assigned to changes in blood flow, including microvascular circulation, as there is no flow at all.

In the analyzed patient groups, atypical courses were quite numerous (about 50%). At the same time, no changes were observed in the FM parameter, which confirms the lack of significant differences in vascular microcirculation between the groups with typical and atypical ischemic responses (Table 1). Atypical courses have also been reported to occur in large numbers among patients with Systemic Lupus Erythematosus (Bogaczewicz et al., 2019), as indicated by the range of observed changes in IR_max_, as well as among patients with diabetic foot ulcers with low prognosis of healing (Los-Stegienta et al., 2021).

It is worth emphasizing that the same factors which in the short term reduce the level of ischemic response, such as strenuous physical exertion, also cause increases in the level of basal fluorescence (Bugaj et al., 2019), indicating a decrease in mitochondrial efficiency as a result of strenuous exertion. On the other hand, intake of inorganic NO_3_^−^ or NO_3_^−^-rich products, such as beetroot juice, through the increase of NO *in vivo*, transiently elevates the level of ischemic response IR_max_ (approx. 30%), while decreasing the level of basal fluorescence (Jurga et al., 2024). This allows us to assign both these effects to the mitochondrial reaction. It has recently been demonstrated that dietary nitrate significantly improves mitochondrial efficiency by improving oxidative phosphorylation efficiency, which reduces oxygen cost during exercise and maximal ATP production (Larsen et al., 2011).

The ischemic response parameter IR_max_ can therefore provide a direct measure of mitochondrial efficiency or dysfunction.

### 3.4 Hyperemic response and reperfusion

After occlusion, the cuff pressure is released and the NADH fluorescence falls below the baseline, reaching a minimum followed by a return to the baseline. This third measurement stage, called the hyperemic response (HR), consists of a very rapid decrease in NADH fluorescence due to hyperemia (20–30 s), followed by a slow return of NADH fluorescence to the baseline due to reperfusion (approximately 3 min).

#### 3.4.1 Vascular dysfunction

The most important parameter characterizing this stage is HR_max_, which is a measure of the maximum decrease in NADH fluorescence after the release of flow in the brachial artery. This decrease occurs as oxygen flows rapidly into the epidermal cells, relative to the resting flow, due to the release of NO and the dilation of the brachial artery. This process can be correlated with the results obtained from FMD or LSCI and mainly concerns the macrocirculation (Gori et al., 2008; Liu et al., 2024; Thijssen et al., 2011). The diagnostic potential of the HR_max_ parameter includes assessment of dysfunction of the macrocirculation, which facilitates assessment of the risk of developing cardiovascular diseases often comorbid with other diseases, such as diabetes mellitus (Hellmann et al., 2017; Katarzynska et al., 2020; Katarzynska et al., 2019a; Tarnawska et al., 2018).

In the case of atypical signal courses with a negative ischemic response (IR_max_ < 0), the HR_max_ parameter cannot be so easily interpreted, because the very rapid hyperemic decrease in NADH fluorescence after the release of arterial flow is perturbed by the changes caused in the NADH/NAD^+^ redox balance by the negative ischemic response. Nevertheless, the slow return of the NADH fluorescence signal to the pre-occlusion baseline level with almost identical kinetics to typical FMSF signals allows for the interpretation of HR_max_ to assess the risk of developing cardiovascular diseases.

#### 3.4.2 Microvascular and mitochondrial functioning

A characteristic feature of FMSF patterns, which appears in most cases at this stage, are clearly visible, strong oscillations of the microcirculation on the reperfusion line. The altered strength and frequency of oscillations (in particular myogenic oscillations (0.052–0.15 Hz)) reflects the reaction of the vascular microcirculation to transient ischemia. The intensity of these myogenic oscillations is called hypoxia sensitivity (HS), because hypoxia is responsible for the increased activity of the vessels after post-occlusive reactive hyperemia. Whereas the HS parameter varies within a very broad range, log (HS) remains normally distributed. The HS parameter, similarly to efficient stabilization of HIF-1α in microvascular smooth muscle cells during transient hypoxia, reflects the microcirculatory response to hypoxia. It allows for assessment of microcirculatory dysfunction in diabetes, cardiovascular disease, peripheral arterial disease, and hypertension, as well as assessment of exercise tolerance (Marcinek et al., 2023).

In patients with impaired ischemic response, a decrease in HS may additionally confirm mitochondrial dysfunction. Growing evidence from recent studies indicates a close connection between hypoxia inducible factor (HIF-1α) stabilization and mitochondria. HIF-1α regulates mitochondrial respiration and oxidative stress. On the other hand, there is evidence supporting mitochondria-triggered HIF-1α activation under various conditions, including hypoxia (Fuhrmann and Brüne, 2017; Huang et al., 2023; Jewell et al., 2001; Kierans and Taylor, 2021; Li et al., 2019; Mialet-Perez and Belaidi, 2024).

It is therefore unsurprising that severe redox imbalance, which is a significant problem occurring in diabetes and its complications, leads to impaired ischemic response (IR_max_) and a lower response to hypoxia, as measured by the HS parameter. The statistically significant reduction in the HS parameter observed in the case of patients with diabetic kidney disease may accompany mitochondrial dysfunction (Los-Stegienta et al., 2022). Low values for the HS parameter were also associated with poor prognosis for healing in difficult-to-heal wounds (including diabetic foot ulcers) (Los-Stegienta et al., 2021). In long COVID patients treated for 3 months with angiotensin-converting enzyme inhibitors and beta-adrenolytics, a statistically significant increase in the HS parameter was observed, with a simultaneous and also statistically significant improvement in the ischemic response IR_max_ (Romanowska-Kocejko et al., 2025).

The correlation between the HS and IR_max_ parameters is more complicated, however. In healthy individuals, a short-term decrease in the IR_max_ (intense exertion) or an increase in the IR_max_ (a single dietary nitrate uptake) is associated with increases or decreases in the intensity of myogenic oscillations, respectively. The microcirculation compensates for changes in the NADH/NAD^+^ balance associated with hypoxia or hyperoxygenation of epidermal cells. Finally, in the case of serious microcirculation disorders, the HS parameter remains very low and does not respond at all to hypoxic conditions causing NADH/NAD^+^ imbalance.

## 4 Conclusion

In conclusion, by interpreting the parameters and the dynamics of the NADH fluorescence signal emitted from skin cells in response to ischemia and subsequent hyperemia, it is possible to identify vascular and mitochondrial dysfunctions that accompany or can lead to the development of chronic diseases. In the FMSF method, decreasing values of flowmotion (FM), hyperemia response (HR_max_), and hypoxia sensitivity (HS) parameters enable the identification of vascular circulation disorders. Decreasing values of ischemia response (IR_max_) and hypoxia sensitivity (HS) parameters, with simultaneous increases in baseline fluorescence (FL_base_), indicate mitochondrial impairment. All the above parameters are weakly negatively correlated with age (Supplementary Table S2), except for baseline fluorescence FL_base_ which increases with age. Differences are also observed between women and men. FL_base_ is higher in women, IR_max_ is lower, and HR_max_ is also higher. The higher FL_base_ and lower IR_max_ values indicate greater susceptibility to mitochondrial dysfunction, while the higher HR_max_ indicates better macrocirculation in women.

Monitoring mitochondrial impairment based on NADH/NAD^+^ balance cannot be performed without simultaneous monitoring of vascular circulation. Dysfunction of one of these factors affects the other. The Flow Mediated Skin Fluorescence method, which uses various parameters to assess vascular circulation and mitochondrial function, allows us to study the feedback between mitochondrial and vascular regulation.

## Data Availability

The original contributions presented in the study are included in the article/Supplementary Material, further inquiries can be directed to the corresponding authors.
