# Supplementary material for: Simultaneous assessment of mitochondrial and vascular function using the Flow Mediated Skin Fluorescence technique
Source: Front Physiol. 2025 Feb 19;16:1509159. doi: 10.3389/fphys.2025.1509159 (PMC11879957; doi:10.3389/fphys.2025.1509159)
Supplement: Supplementary file 1 [file DataSheet1.pdf]

Supplementary Material

1. Description of the analyzed groups

All study participants were over 40 years old and most patients were overweight or obese.

Clinical characteristics of CVD patients: age, 69.4 ± 10.7 years; male/female ratio, 218/264; Body Mass Index (BMI), 29.4 ± 5.9; Systolic Blood Pressure/ Diastolic Blood Pressure (SBP/ DBP), 139.1/ 74.4 ± 18.0/ 10.6; cardiovascular co-morbidities, 337 (70 %); diabetes, 159 (33 %).

Clinical characteristics of patients with DM2: age, 63.0 ± 8.1 years; male/female ratio, 38/32; HbA1c (%), 8.2 ± 1.4; disease duration, 14.6 ± 7.4; Body Mass Index (BMI), 31.4 ± 5.5; Systolic Blood Pressure/ Diastolic Blood Pressure (SBP/ DBP), 134.6/ 78.2 ± 13.6/ 9.2.

2 Supplementary Tables

Statistical analyses were performed with OriginPro 2023 software.

Table 1S. Characteristics of the study groups by sex. Results are shown as mean ± standard deviation SD) with corresponding *p*-value by unpaired Student's *t*-test or Mann–Whitney test as appropriate. Only statistically significant differences were indicated ($p < 0.05$).

CVD (N = 482)	**All**	**Female**	**Male**	***p-value*** **(female vs male)**
**N**	482	264	218	
**Age [years]**	69.4 ± 10.7	70.4 ± 10.8	68.1 ± 10.4	0.015[a]
**BMI [kg/m^2]**	29.4 ± 5.9	29.6 ± 6.0	29.1 ± 5.9	
**$FL_{base} \times 10^3$ [a.u.]**	483 ± 206	532 ± 196	423 ± 203	<0.0001[a]
**log(FM)**	1.33 ± 0.40	1.34 ± 0.38	1.31 ± 0.42	
**IR_{max} [%]**	11.2 ± 5.7	10.6 ± 6.0	11.8 ± 5.3	0.024[a]
**HR_{max} [%]**	16.3 ± 5.1	17.3 ± 5.0	15.1 ± 5.0	<0.0001[a]
**log(HS)**	1.10 ± 0.55	1.11 ± 0.53	1.07 ± 0.56	

[a]Mann-Whitney test
Abbreviations: BMI, Body Mass Index; FL_{base}, Fluorescence at baseline; log(FM), FlowMotion at baseline (logarithm); IR_{max}, Ischemic Response; HR_{max}, Hyperemic Response; log(HS), Hypoxia Sensitivity (logarithm).

Table 2S. Correlations of FL_{base}, log(FM), IR_{max}, HR_{max}, RHR and log(HS) parameters with age in the CVD group. Results are shown as Pearson or Spearman (as appropriate) correlation coefficient (*r*) and *p* value (*p*). Statistical significance was set at $p < 0.05$.

CVD (N = 482)		**FL_{base}**	**log(FM)**	**IR_{max}**	**HR_{max}**	**log(HS)**
**Age**	*r*	0.118[a]	-0.140[b]	-0.233[a]	-0.090[b]	-0.168[a]
	p	0.009	0.002	<0.0001	0.048	0.0002

[a]Spearman Corr.; [b]Pearson Corr.

Abbreviations: FL_{base}, Fluorescence at baseline; log(FM), FlowMotion at baseline (logarithm); IR_{max}, Ischemic Response; HR_{max}, Hyperemic Response; log(HS), Hypoxia Sensitivity (logarithm).
